# Recovered but Constrained: Narratives of Ghanaian COVID-19 Survivors Experiences and Coping Pathways of Stigma, Discrimination, Social Exclusion and Their Sequels

**DOI:** 10.34172/ijhpm.2021.81

**Published:** 2021-08-09

**Authors:** Roger A. Atinga, Nafisa Mummy Issifu Alhassan, Alice Ayawine

**Affiliations:** ^1^Department of Public Administration and Health Services Management, University of Ghana Business School, Accra, Ghana.; ^2^Ghana Health Service, Abokobi Health Centre, Accra, Ghana.; ^3^Faculty of Health and Allied Sciences, Catholic University College of Ghana, Sunyani, Ghana.

**Keywords:** Stigmatization, Discrimination, Social Exclusion, COVID-19, Coping Strategy, Ghana

## Abstract

**Background:** Research about the coronavirus disease 2019 (COVID-19), its epidemiology and socio-economic impact on populations worldwide has gained attention. However, there is dearth of empirical knowledge in low- and middle-income settings about the pandemic’s impact on survivors, particularly the tension of their everyday life arising from the experiences and consequences of stigma, discrimination and social exclusion, and how they cope with these behavioral adversities.

**Methods:** Realist qualitative approach drawing data from people clinically diagnosed positive of COVID-19, admitted into therapy in a designated treatment facility, and subsequently recovered and discharged for or without follow-up domiciliary care. In-depth interviews were conducted by maintaining a code book for identifying and documenting thematic categories in a progression leading to thematic saturation with 45 participants. Data were transcribed and coded deductively for broad themes at the start before systematically nesting emerging themes into the broad ones with the aid of NVivo 12 software.

**Results:** Everyday lived experiences of the participants were disrupted with acts of indirect stigmatization (against relatives and family members), direct stigmatization (labeling, prejudices and stereotyping), barriers to realizing full social life and discriminatory behaviors across socio-ecological structures (workplace, community, family, and social institutions). These behavioral adversities were associated with self-reported poor health, anxiety and psychological disorders, and frustrations among others. Consequently, supplicatory prayers, societal and organizational withdrawal, aggressive behaviors, supportive counseling, and self-assertive behaviors were adopted to cope and modify the adverse behaviors driven by misinformation and fearful perceptions of the COVID-19 and its contagious proportions.

**Conclusion:** In the face of the analysis, social campaigns and dissemination of toolkits that can trigger behavior change and responsible behaviors toward COVID-19 survivors are proposed to be implemented by health stakeholders, policy and decision makers in partnership with social influencers, the media, and telecoms.

## Background

**Key Messages**

** Implications for policy makers**

- This study contributes to enhancing understanding of the many ways in which coronavirus disease 2019 (COVID-19) has disrupted not only health systems but also the lived experience of persons infected with the disease.
- Stigma, discrimination and social exclusion against COVID-19 survivors originated from the individual, societal and organizational levels. The findings lay the foundation for tackling behavioral adversities across these socio-ecological levels.
- Health policy and decision makers should collaborate with social influencers to launch social campaigns targeting at averting stigma and discrimination against COVID-19 survivors. By so doing, society will become emotionally tuned to COVID-19 survivors by embracing them within the family and community structures.
- Behavior change role plays with inscriptions such as “who are you to judge” have profoundly diffused stigma and discrimination against key populations and can equally be deployed to eliminate labelling, prejudices, stereotyping and social disapprovals encountered by COVID-19 survivors.
- Psychosocial support services are urgently needed to rebuild broken minds, thoughts and disorientations brought about by routine experience of stigma and discrimination against survivors and their family and relatives.

** Implications for the public**

The narrative from existing literature has not been explicit on how and why coronavirus disease 2019 (COVID-19) survivors experience and cope with stigma, discrimination, social exclusion and their sequels. By deploying qualitative methods, this study showed how everyday lived experiences of COVID-19 survivors were disrupted in many unfortunate ways. They were socially discredited and regarded as ‘unpleasant for contact’ by the society. Prejudices, stereotypical behaviors and labelling directed at participants weakened their morale, happiness and self-worth while discrimination was associated with distress, frustration and psychosocial problems. The findings, overall, highlight how the survivors were constrained as they seemingly faced a “double pandemic” of battling for physical recovery in addition to fighting stigma and discrimination within the societal and organizational context. Systematic interventions targeting preventing misinformation and fear that triggers stigma, discrimination and social disapproval of post-infected persons is urgently needed.

The global spontaneous transmission of the severe acute respiratory syndrome coronavirus 2 (SARS-CoV-2) also known as coronavirus disease 2019 (COVID-19) is having an intolerable burden on all aspects of human lives and livelihood, health systems functioning and global health security. Health logistics and supply chain systems have been greatly disturbed, vulnerable populations face care access constraints, hospitals are outnumbered with increased admissions, and health workers endure enormous burnout, job hazards and psychological challenges.^1^ Within the general population, mitigation approaches such as lockdowns, wearing of face mask and social distancing are causing fear, worries, insecurity and psychological distress which increases vulnerability to health risks.^2-4^

 The regressive impact of the pandemic is felt more on survivors within the general population who experience post-infection stigma and discrimination^5^ leading to socio-economic adversities and maladaptation to homes, communities, and work settings. Stigma and discrimination against COVID-19 survivors have been particularly highlighted in the literature because of the many different shades of perceptions, understanding and knowledge of the disease epidemiology across settings.^6^ Stigma and discrimination against persons infected with the COVID-19 is a global concern because of their consequences for poor physical, mental health and social wellbeing.^7,8^ Negative beliefs, attitudes and perceptions held against COVID-19 infected populations destabilizes therapeutic responses leading to poor recovery efforts and inevitable health complications in the short to long term.^7^ In light of this, Peprah and Gyasi^9^ echoed that rising stigma and discriminatory behaviors against COVID-19 survivors especially in the sub-Saharan Africa context impedes progress on the fight against the pandemic which calls for concerted averting actions by relevant health stakeholders and policy-makers.

 In Ghana, the COVID-19 pandemic has accumulated diverse social constructions and narratives in many contexts and cultural spaces. The pandemic is perceived and understood across some contexts as a mysterious epidemic, divine punishment to atone human guilt, divine cleansing spirit of Mother Earth or a hoax, similar to views held in other settings.^10,11^ The delay in the discovery of treatment vaccine for the disease coupled with the volume of ‘information noise’ and fake news^6^ tends to amplify the misperceptions that are being embraced, sustained, and profoundly diffused through informal channels. In some communities, questions about how, why, and under what circumstance a person gets infected with the COVID-19 invoke answers embedded in norms, beliefs, cultures, and social constructions of the pandemic, rather than the prevailing biomedical viewpoints.^6^

 The multiple interpretations, misconceptions and rumors surrounding the COVID-19 pandemic and its transmission mode is having perverse effect in the way people perceive, approach, and behave toward survivors. COVID-19 survivors and their families are reported to face discrimination, rejection, stigmatization and isolation, all of which are driven by fear of being infected and fear-induced misrepresentations and misinformation about the pandemic within cultural, organizational, religious and social circles.^6^ In some instances, individuals clinically diagnosed positive, treated and tested negative are perceived to be permanently infected and face social avoidance within the immediate and structural environment.^12^ As a result, persons with symptoms are often reluctant to get tested, because of the risk of being exposed to public prejudicial reactions, humiliation, and other social derogatory quips. Against this background, survivors of COVID-19 could suffer post-infection syndromes of depression, mood disorder, mental health challenges, and psychosocial problems without appropriate evidence to inform decisions about interventions to manage stigma, discrimination, and adverse social behaviors.^13^ Unresolved post-infection sequela are tipping points for a spectrum of clinical syndromes including cognitive, neurological, and traumatic disorders as well as long-term depression leading to premature mortalities.^5,14^

 As more populations are being infected with the COVID-19, gaining deeper understanding of the experiences of unjust treatment, challenges, changing disposition and relational orientations, and negative perceptions held around COVID-19 survivors is urgent and imperative to inform the design of mitigation strategies to alleviate debilitating effects. Yet studies about stigma and discrimination have not gained significant empirical attention especially in sub-Saharan Africa including Ghana which accounts for 2.04% of confirmed COVID-19 cases in the Continent as of March, 2021.^15^ A range of published studies focusing on stigma and discrimination against COVID-19 infected persons other than frontline health workers have been largely drawn on reviews of cross cutting literature^9,16,17^ which are more generic in scope and limit application to context specific circumstances. Moreover, how stigma and discrimination are linked to social exclusion and the resulting consequences on disruptions to socio-economic, physical and mental wellbeing of COVID-19 survivors has been poorly explored empirically. Drawing strength from the literature gaps, we deployed qualitative methods to explore the lived experiences of COVID-19 survivors, with particular attention to how and why they are exposed to, and cope with discrimination, stigmatization, and social exclusion from the immediate and wider environments. How these disrupting experiences impair physical, psychosocial and socio-economic well-being are also explored.

## Methods

###  Study Context 

 The study was conducted among post infected COVID-19 patients in the Greater Accra Region (GAR) of Ghana. At the time of the study, the GAR had the highest reproduction number, and topped all other regions with the numbers of active and cumulative COVID-19 confirmed cases (Figure).^18^ The GAR account for more than 80% of active and cumulative cases in the country, because it host the capital and largest commercial city where dense economic, social and political activities intersect in many ways. Moreover, the existence of a large informal economy, a high population density, and urban residential trajectories – poorly planned, congested and downgraded neighborhoods have practically constrained efforts at successful implementation of, and adherence to, the COVID-19 mitigation protocols.

**Figure:**
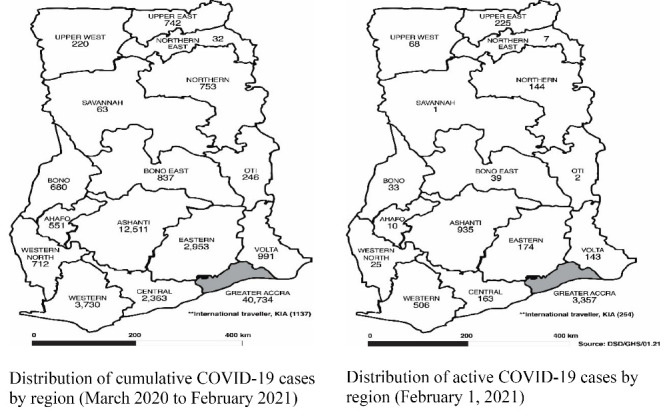
National Distribution of Active and Cumulative COVID-19 Cases. Abbreviation: COVID-19, coronavirus disease 2019. Source: Ghana Health Service routine COVID-19 updates.

 To mitigate the rate of transmission in the region and the entire country, the government established COVID-19 testing and treatment facilities equipped with ventilator support services, beds, personal protective equipment and other essential medical tools for managing COVID-19 cases with severe symptoms on admission. In addition, several medical officers, nurses, paramedics and other clinicians have been trained to provide in-person clinical services, care plans, counselling services, medication management and domiciliary follow-up care to COVID-19 patients in the safest way possible.

###  Participants Recruitment and Interview

 Realist qualitative approach was used to explore how and why COVID-19 survivors experience stigma and discrimination, the resulting consequences and the coping mechanisms that become embedded in their routine personal and social functions. A survivor was defined as a person clinically diagnosed positive of COVID-19, admitted into therapy in a designated treatment facility, and subsequently recovered and discharged for or without follow-up domiciliary care. Participants were recruited through contacts with the COVID-19 case management team (medical officers, nurses, clinical psychologist, and paramedics) working in the treatment facilities. The research team comprised of the first 2 authors and 3 research assistants who were given a day’s training on the content of the interview tool, approaches to gathering qualitative data, recording techniques and data handling. The first author contacted the Heads of the treatment facilities and explained the study’s purpose, objectives, potential usefulness of the findings, the targeted participants, and then sought their approval. Upon given permission, the research team worked with the data managers of the facilities to recruit participants. Using the clinical records, we extracted the phone numbers and basic demographic information of survivors who were 18 years or older and without clinical complications with debilitation effect. Names, addresses, and any form of identity of the survivors were masked from the research team.

 About 1213 valid phone numbers of the targeted participants were extracted. However, 128 (22.2%) of the 576 participants successfully contacted agreed to participate in the study. The rest cited disinterest, poor physical fitness, and unavailability among others as reasons for opting out. To ensure safety of the participants and the research team, telephone interviews were conducted in agreement with each participant. The interviews were call recorded and retrieved via the call recorder app for android and iPhones. Zoom and Skype interviews were held with 8 participants who requested for such mediums. To prevent digital intrusions that potentially compromise anonymity of participants in the online interviews, the recordings were set to be retrieved via the personal computer and subsequently passworded. The telephone and online recording of the interviews were complemented by note taking of key issues emerging as the interviews proceeded.

###  Data Collection Tools and Approaches 

 Data were sourced using an interview guide consisting of 6 broad thematic areas: (*a*) participant’s knowledge of how they acquired the virus and the reactions from the family, friends and the wider community after testing positive; (*b*) experience of discrimination and stigmatization from the workplace and community; (*c*) experience of changing disposition and social relations; (*d*) the difficulties if any of integration into the family, community and workplace after recovery; (*e*) coping strategies devised in response to stigma and discrimination; and (*f*) the impact of these experiences on the physical, psychosocial and mental well-being.

 The interviews were more of conversations allowing participants to direct the course as much as possible. Rather than mechanically following the sequence of questions fashioned in the interview tool, the use of inductive probes and the resulting responses directed much of the conversations. About 85% of the interviews were conducted in English. The rest were held either in Ga or Twi (local languages widely spoken in the Region).

 We followed the thematic saturation method earlier used by Guest et al^19^ by maintaining a code book for identifying and documenting thematic categories in a progression. The code book was routinely monitored and updated to accommodate emerging ones as the interviews progressed. For example, during the code book monitoring process, we observed interesting developments in participants narratives about how they got infected and the personal reaction after testing positive. Furthermore, 3 of the first 5 participants were assertive that their families were victims of stigmatization within the community. These emerging themes were thus, integrated into the code book and explored further (Table 1). When new interview data produced little or no significant change to an existing category, that category was deemphasized in the subsequent interview proceedings. This process continued until we obtained thematic exhaustion and data variability from 45 participants.

**Table 1:** Code Book of the Original and Emerging Themes

**Conceptual Theme **	**Description **	**Initial Themes**	**Emerging Themes **	**No. of Times Mentioned**
Stigmatization	Social disapproval of a person by virtue of their infection	Prejudices Stereotyping Undermining status	Labelling Stigma against family/relatives	39
Discrimination	Unjust or unfair treatment against an infected person	Unfair treatment at work and community	Mistreatment by family Discrimination in access and use of public facilities	31
Social exclusion	Avoidance or rejection of an infected person within the social environment	Social avoidance Barriers to social adjustment	Adverse social behaviors Narrow social spaces	21
Infection history	Participants’ recount of how they got infected with the COVID-19	Source of infection Adherence to protocol at the time of infection		41
Reaction to test outcome	Response after receiving positive test results	Psychosocial responses Emotional response	Suicidal intention Fear Traumatize Dejection	33
Sequels of the adverse experiences	Consequences of experiencing stigma, discrimination, and social exclusion	Psychosocial disorder Anxiety disorder Distress	Demoralized Feeling worthless Loneliness Frustration Withdrawal from friends Confusion	39
Coping strategy	Mechanisms of managing stigma, discrimination, social exclusion and their sequela	Looking up to God with prayers Relying on family and relatives	Believing in self Societal/organizational support Avoiding social relations Aggressive behavior	36

Abbreviation: COVID-19, coronavirus disease 2019.

###  Analysis 

 To accurately capture the detail full length of the recorded data, the second author and 1 of the research assistants independently transcribed all the audio recordings verbatim in English. The pairs of transcripts were then reviewed and refined for clarity and reconciled jointly by the authors. Building on the code book structure earlier adopted, the first author and an independent person with expertise in qualitative data analysis developed broad themes reflecting key conceptual issues, and then iteratively worked through the transcripts to generate and nest, sub-themes into the broad categories, and basic codes into the sub-categories using the NVivo 12 software. In working our way into the themes building process, we identified and mapped thematic typologies into a cause-and-effect configuration using tables. Demographic characteristics are presented with descriptive statistics computed using Microsoft Excel.

## Results

 Demographic characteristics of the participants are shown in Table 2. Most of the participants were males (64.4%), had basic/senior high education (46.7%) and self-employed (42.2%). The mean age was about 38 years.

**Table 2:** Demographic Information of Participants

**Characteristic **	**Description **	**No. **	**%**
Gender	Male	29	64.4
	Female	16	35.6
Age	Mean	37.7	
	Standard deviation	16.9	
	Range	28-69	
Education	None	7	15.6
	Basic/senior high	21	46.7
	Higher	17	37.8
Occupation	Self-employed	19	42.2
	Employed in public sector	15	33.3
	Employed in private sector	11	24.4

 A total of 8 main themes, 11 sub-themes and 54 basic themes were derived from the analysis and coding process (Table 3). The findings are structured and presented in sections according to the main thematic areas. In some of the sections, sub-themes developed are discussed together with the basic themes supported by verbatim quotes.

**Table 3:** Major, Sub and Basic Themes Derived From the Coding Process

**Main Themes **	**Sub-themes **	**Basic Themes **
Infection dilemma and consequences of prolonged wait for test results		Source of infection Adherence to COVID-19 protocol at the time of infection Testing and time lag in the receipt of test results
Reaction after receiving test results showing positive		Mode of transmission of test results Physical and emotional reaction to positive test results Post-infection shocks
Experiences of stigmatization	Social stigmatization	Prejudices Stereotyping behaviors Emotionally charged labels
	Socially undermining behaviors	Loss of respect Modification of public perceptions
	Stigmatization against family and relatives	Avoidance of family/relative occupation Family removal from social contacts in the neighborhood Negative perceptions against family members
	Cultural elements and stigmatization	Traditional rituals for protection against COVID-19 Cultural interpretations of infection causing stigmatization
Discrimination in the family and community	Discrimination within the family structure	Discriminatory behaviors of family members Poor attention given to participants Literally victimized for being infected
	Restrictions in access and use of public facilities	Denial of access to community public facilities and recreational areas
Workplace discrimination	Unfair treatment	Unfair treatment by immediate supervisor Denial of opportunities at the workplace Negative orientations
	Modification in relational attitudes and orientation	Mood changes toward participants Unwelcoming attitude Relational barriers at the workplace
	Functional discrimination at work	Denial from the use of shared office tools and equipment Limited access to full complement office space
Social exclusion	Barriers to social life	Perceived threat to social order Limited access to social spaces
	Socially avoiding behaviors	Social neglect Social segregation
Sequels of the adverse experiences		Diminished happiness Low morale and self-confidence Reduced self-worth Frustration Worries Anxiety disorders Delayed recovery Distress Loneliness Fear Social withdrawal Insomnia
Coping mechanisms		Relying on social support systems from family/friends Believing in self Assertiveness Supplicatory prayers Supportive counseling Withdrawal from friends and society Aggressive behaviors Distancing behavior Poor commitment to work Organizational withdrawal

Abbreviation: COVID-19, coronavirus disease 2019.

###  The Infection Dilemma, Prolonged Wait for Test Results and the Consequences 

 Almost all the participants expressed dilemma as to how they got infected with the virus. They were able to some extent narrate their history of travels and mingling in religious congregations, social events (eg, wedding and outdooring ceremonies, funerals etc) and economic activities (eg, going to market squares, shopping centers etc), but were skeptical that such events were the source of infection. To some participants, the source of infection was more or less a puzzle (“to date am still thinking about how I got infected;” “I am not sure where I probably got infected;” and “my infection is still a mystery”). Most participants admitted to partial or no adherence to the COVID-19 protocols at the time of infection because of misinformation and rumors that the COVID-19 is “a white man disease,” “blacks have strong immunity to COVID-19” and “COVID-19 is a deception to make money from sales of personal protection equipment.” Wearing of face mask was perceived as discomforting and dangerous to human health as reflected in opinions like:

*“I will not cover my nose and die because of a foreign sickness imported to my country.” *

*“Face mask suffocates, why should I wear something that can cause my death.” *

 The data were riddled with stories of preventable infections complicated by delay in being tested and receipt of test results leading to further infections. This was exemplified in excerpts from some participants’ narratives. For example, we learned from the intricating story of a health worker (nurse) who tested positive owing to her own negligence (Box 1).

**Box 1. Narrative of a Health Worker Who Tested Positive Owning to Negligence**

I was infected by a colleague nurse who travelled and missed the mandatory test organized by the facility in which everyone was tested negative. My routines at the workplace were tied to her. We used to eat together, chitchat and hold clinical sessions together. But the problem was that we were both not strictly adhering to the COVID-19 protocols, we took things for granted. She was also pregnant at the time, so I decided to help her during working hours. Within some few days, I realized she started showing progressive respiratory distress with severe cough and abnormal full blood count after lab investigation was conducted. I got scared, very scared, and asked her to go for COVID-19 test. She initially objected but later agreed. When the test result came, it was positive. This increased my fright. I also took the test, and it was positive. In my case, the test results delayed after the sample was taken. So I thought everything was fine with me because I only had mild catarrh and sometimes slight headache. Ten days later, my results came positive. I did not isolate, and three colleague workers were infected by me.

Abbreviation: COVID-19, coronavirus disease 2019.

When asked why she did not self-isolate pending outcome of the result, she replied:

*“Self-isolation was the last I could think of because there were no serious symptoms. I had mild cold and cough unlike my colleague, so I felt it was normal. I later felt sorry for infecting my colleagues.” *

 Another participant recounted how his closed relative showed symptoms of cough, cold and respiratory disorders but failed to take the test despite persistent advice because of a personal conviction that it was normal respiratory tract infection. For this reason, they kept to their communal life and casual relational mechanisms – shaking of hands, hugs, close chat and more without regards to the protocols. He suspected being infected after experiencing severe cough, shortness of breath and rendered himself for testing. The result which turned out positive was produced in seven days during which he had infected other relatives and people in the workplace.

###  Reaction After Receiving Results Showing Positive 

 Test results were transmitted to most participants through phone calls and text messaging. The absence of prior counseling services to prepare minds and allay fears before disclosure of positive test results prompted adverse reactions borne out of the many mysteries, rumors and wrong perceptions about the COVID-19 and its mortality proportions. Thoughts of dying from the COVID-19 weakened participants physically and mentally as many reported fear, anxiety, worries and traumatic disorders when the test result was disclosed to them.

*“I was traumatized. You know it is a killer disease and many have died, so since I also got it, I felt traumatized. Since then, I get frightened when I hear corona virus.” *

*“…I could not hold myself. I was experiencing mood disorders because of information that Covid is deadly. In fact, for some days I thought I was not worth living again.” *

 News of the positive test results dealt a fatal blow to some participants who felt aggrieved and devastated while others developed suicidal ideations because dying from COVID-19 was not dignifying.

*“When I heard the results of my test, I was actually thinking about ending my life. It is better to do so that than dying from corona virus where they will bury you anyhow.” *

 At the time of the study when participants were recovering, shocks of infection with the COVID-19 were still apparent. This was exemplified in some participants lamentations and outpouring snivel that they were experiencing reverse life. Routine experience of stigma, discrimination and social relational barriers compounded their predicaments. These are further explored below.

###  Experiences of Stigmatization 

####  Social Stigmatization 

 Prejudices linked to social stigmatization was widely reported. A cross section of the participants reported experiencing sudden social disapproval within the community. Socially unwelcoming behaviors, cold mood and negative perceptions toward participants were reported. A participant explained how she was suddenly being viewed differently from the rest of her neighborhood and subjected to persistent awkward gawking in public:

*“Too often I come across people who would just be staring at me and when I turn, they look away. It is all because they know I have been tested positive for the corona virus.” *

 Social stigma also took the form of negative stereotyping (“they say I would never be strong again” and “I am a health threat”) and labelling with all manners of offensive descriptions. Some participants said they received emotionally charged labels and derisive comments which were demoralizing and frustrating:

*“It is painful when someone knows your name and jokingly calls you Mr. Corona. I get frustrated by that.” *

*“…even people I know who should be protecting and supporting me call me Coro Coro. It is sad and very demeaning.” *

####  Socially Undermining Behaviors 

 Linked but distant from stereotyping, were socially undermining behaviors that purportedly discouraged participants in many ways. The first was loss of respect because elements of the labelling made participants less worthy of attention. Second, the fact that participants were perceived as health threat dented their reputation and decision space. A health provider who prior to infection had voluntarily carried out public education on prevention of the COVID-19 explained how individuals negatively evaluated and modified perceptions about him.

*“…I was explaining to them that they should follow the protocols careful (….) I also said I was more than careful yet I got infected. Then I could hear comments like upon all your knowledge you still got it.”*

####  Associated Stigmatization Against Family and Relatives 

*“She used to sell more than 500 Ghana cedis a day, but for a month now she will just open the shop and close without making sales. Nobody comes to buy because of me. Everyday perishable items like bread go bad and have to be thrown away.” *

 The above quote characterizes the experience of a participant whose immediate and extended family members were stigmatized to the extent that the community avoided a grocery shop belonging to the sister. The shop attracted significant labelling as “Corona Shop” or “Coro Shop.” Existing and potential customers avoid the grocery shop because the widespread ridicule induced fear of infection:

*“People are afraid to buy from the shop. Even if people are going to the shop, they will just tell them that beware it is a corona store.” *

 Another participant recounted how his family was stigmatized and removed from social contacts within the neighborhood because of deluding information that “his family is not safe.” This stigma was further heightened by the apparent diffusion of falsehood information that his entire family tested positive and that anyone in contact with the family risk infection. He further explained that nearby retail shops avoided and rejected his family in need of basic necessities of life.

*“My children have been driven away from shops around countless times, for apparent reasons, because I got infected. Look, my children will tell me they see the items they want in the shelves yet shop attendants will pretend such items are out of stock.” *

####  How Cultural Elements Fueled Stigmatization 

 The data suggested that cultural undertones intersected with fear of infection to compound stigmatization against families in some communities. Some participants revealed that when COVID-19 was first reported in Ghana, elders of the community performed traditional sacrifices, libation and purifications ostensibly to invoke the gods’ intervention and spiritual protection of the community against the COVID-19. The belief formed, following such traditional practices was that everyone from the community was immune to the virus. Thus, their infection, which was a rare occurrence, generated waves of doubts, uncertainties and cultural interpretations that heightened the stigmatization.

*“The moment people got to know that I have COVID-19, they couldn’t believe it. They said I have offended the gods, they are scared and that is why people avoid me and my family.” *

###  Experiences of Discrimination 

####  Discrimination Within the Family Structure 

 Of the 10 participants who reported experiencing discrimination within the family, one participants’ narrative was most profound. A graduate who was posted for national service at a public institution said he was tested positive following contact tracing. When he disclosed the status to the family there was panic reaction from everyone. He was suddenly met with discriminatory behaviors including social avoidance, deprivation in the use of household items and exclusion from the family system. He was literarily victimized for his condition and eventually asked to leave the house and rent accommodation elsewhere in order not to infect other members of the family. Sensing that his meagre national service allowance of about GHS 500 was insufficient to secure accommodation on time to move out of the house, his father supported him to rent a room that cost GHS 4000 (US$: 696.86; US$ 1 = GHS 5.74). Another participant said he endured unreceptive behavior and poor attention after refusing a family order to leave the house.

####  Restrictions in Access and Use of Public Facilities 

 Some participants complained that although they had recovered, they were still denied access to community public goods such as toilet facilities, parks, and recreational areas. A participant was refused access to a public community lavatory on grounds that she would infect others. While leaving in search of an alternative place to use, the lavatory attendant sarcastically remarked that “we don’t sell corona here.” Other users queuing for the facility also avoided the place momentarily and returned when she was gone. Another participant who masked and stood solitary watching a community amateur football match was approached by two people and asked to leave the field because “he poses health risk to the footballers and the sparsely gathered spectators.”

###  Discrimination at the Workplace 

####  Elements of Unfair Treatment 

 Of the forms of workplace discrimination observed, unfair treatment was more pronounced because its manifestation cut across different sectors of the labor economy. Two participants pointed to being treated unfairly by their immediate managers when they reported to work after testing negative. One of them was denied the opportunity to participate in a mandatory staff meeting because “everyone at the workplace was told to be careful with him.” The other said although he showed the results indicating negative to his manager, he was still ordered to leave the workplace immediately and report back when called.

*“When I reported to work, I realized my colleagues were staring at me. I met my Boss and told her that I am now okay because I tested negative. She just shouted go! go! go! We will call you when you are needed.” *

 A participant reported that “they were all manners of negative rumors at the workplace about me when I tested positive.” Because of that keys were produced for everyone at the workplace to access the staff lavatory. When she requested for a copy of the key to produce one for herself, her colleague replied that “the In-Charge instructed us not to give the key to anyone.” At this point, she felt discriminated against and that affected her commitment to work:

*“I felt bad when my colleague told me the key is not for everyone as if I am not a staff. Before I got Covid they didn’t treat me that way so why now? Thoughts of that has changed the way I used to give my all to the work.” *

####  Change in Relational Attitudes and Orientation 

 The data showed change in mood, unwelcoming attitudes and relational barriers with participants at the workplace. Those in formal employment were assertive that they were often dissociated from social interactions and collegiality that characterize the workplace. A public sector worker said he moved out of office fully masked and met three colleague workers having a casual conversation. As he approached them to greet, two of them dashed out of the scene while the other pulled out and wore a double face mask before interacting with him briefly. For him, that was the most embarrassing and humiliating moment of his working life.

 The negative orientation about COVID-19 also shaped relational dispositions with participants. A seemingly widespread perception was that individuals who tested positive of COVID-19 were a threat to workplace health and safety. This perception produced a range of behavioral odd relationships with participants such as “they said I will infect them so they try to stay away from me.” and “what goes around comes around, they will also get it and see,” and as illustrated in these statements:

*“Sometimes you are talking to a colleague and he will be looking away. He wouldn’t want to see your face (…) some too will stand at a far distance and be talking to you. I have faced this several times although my mask is always on.” *

*“My colleague workers used to relate nicely with me. But the situation is different when I came back to work.” *

####  Functional Discrimination at Work 

 Some participants were functionally constrained in the performance of their duties after resuming work. A participant was prevented from using shared office tools and equipment like computers, printers and copiers because “he will put others at risk.” Similarly, despite showing the immediate supervisor proof of negative test results, a participant was still restricted from using full complements of the office space: “he said I should sit at one place and avoid touching things around.” In another dramatic fashion, a participant joined colleague workers in search for official documents reportedly missing in the office. When he found the documents, his manager murmured that they were no longer needed. The manager later returned fully covered in a face shield and mask, requested for the documents, sanitized them thoroughly and placed them in a locker cabinet.

*“It was funny how he pretended the documents were not needed all because I was holding them. If it was coming from any of my colleagues, he would have collected. They still believe I can infect them although I am fine, and for how long will this go on?” *

###  Social Exclusion 

####  Barriers to Social Life 

 Participants were perceived as a threat to social order. In Ghana where social life within the family, neighborhood and community intersects in many ways, such negative perceptions encouraged and reinforced social exclusion of COVID-19 survivors from social congregations, events and traditional ceremonies. Social barriers were reportedly created in some communities ostensibly to limit and control access to social spaces. One notable example was when a participant was forcefully withdrawn from a community keep fit and fun walk event involving about 40 partakers. Although she had fully recovered to participate in the fitness club as a member, the notion was that she is still a carrier and therefore potentially dangerous to the health of others.

 Children equally endured social exclusion from their family neighborhoods. A participant noted that on several occasions, her 3 children were prevented from playing with their peers in a community playground. Her neighbors also sternly instructed their children to avoid all her kids who were described as “unsafe to play with.” Consequently, the children were often confined to the home.

####  Socially Avoiding Behaviors 

 A handful of the participants lamented about experiencing social neglect and change in social and relational attitudes from the circle of friends who feared being infected. This narrative is worth pointing out:

*“My experience with a friend at Madina (a suburb in Accra) shocked me. I was sitting at a phone service shop and a colleague saw me, parked his car and then approached me and said I have not been seeing you for some time, what could be the problem. In a very concerned manner, I told him that I was diagnosed with Covid. The moment I said that,, he immediately put on his face mask and said see you again.” *

 This participant’s experience also epitomizes social neglect from friends.

*“One morning I met a friend on my way to work. When I saw her, I was very happy so I called her. But as I was smiling and going closer to her, she pretended she didn’t know me and sort of walked away in a hurry. That day I felt bad! I entered a nearby washroom, wept for some minutes, wash my face and let it go.” *

###  Sequels of the Adverse Experiences of Stigma, Discrimination, and Social Exclusion 

 Elements of the social stigmatization produced ripple effects. First, stigmatization in all forms was shown to diminish happiness, morale and self-confidence leading to the feeling of reduced self-worth. Prejudices and stereotypes were the most disturbing as they routinely frustrated and caused worry and anxiety disorders among participants. At the time of the study, some participants were reportedly hospitalized for treatment of sicknesses related to repeated stigmatization. Delay in full recovery on the part of some participants was also the consequence of social stigmatization. Discrimination from family and friendship circles demonstrated poor love and care for survivors. This resulted in distress, loneliness, and withdrawal from social interaction with family and friends. These are highlighted in Table 4. Flashbacks of the adverse experiences of stigmatization, discrimination and social exclusion interrupted routine activities, as pointed out by this participant.

*“Sometimes I get knocked down by these negative comments and unable to do anything. I do not feel life again.” *

**Table 4:** Sequels and Illustrative Quotes of the Adverse Experience of Stigma, Discrimination, and Social Exclusion

**Adverse Experience **	**Sequel **	**Supporting Quote **
Stigmatization	Social stigmatization weakened participants health, happiness, morale and self-confidence.	*“I am not happy with how people see me negatively. It makes my blood pressure go high*.*”*
	Prejudices and stereotypes were reported to produce frustration, anxiety disorders and insomnia in affected participants.	*“I get worried all the time because of what they think and say about me*.*”* *“The stigma against my sister’s business is worrying me. Any small thing irritates me which wasn’t the case. I felt very sick and reported to a doctor and he said I sleep poorly which is true. I can’t sleep over the issue*.*” *
Discrimination	Participants who experienced family discrimination self-reported distress, dejection, loneliness, and delay in recovery.	*“I feel sad and lonely that there is no love from the family*.*” * *“…sad, very sad is the feeling around me that even my family treated me poorly*.* I try to overcome it, but it is not working*.*” * *“…It is taking me time to recover from the virus because I am depressed with the treatment by my family members*.*”*
	Participants developed fear, eating disorder, psychological problems and frustration from workplace discrimination.	*“I am in shock of everything that go on around me at the workplace. I get scared when I see my colleagues. I can’t even eat well*.*”* *“…their behavior is frustrating me. I feel insecure all the time*.*” * *“I am psychologically struggling to cope at the workplace*.*”*
Social exclusion	Consequences of the social barriers and modified social relations reflected in the experience of anthropophobia.	*“I fear my friends, I fear them. From the look of things, they can say or do anything to harm you*.*”* *“…I don’t want to have anything doing with any friend*.*”*

###  Coping With the Stigma, Discrimination and Socially Undesirable Behaviors 

 The coping mechanisms deployed to overcome the adverse experiences were diverse. Some participants described how they were self-assured and assertive of overcoming the inherent experiences of stigma and discrimination. There was a strong intuition that COVID-19 and its associated acts of stigma and discrimination are only temporal sways of hope and self-worth. By being assertive and believing in oneself, it is possible to challenge the circumstance, misguided beliefs and develop crystal opposing thoughts for peace of mind.

*“If you put your mind in these negative comments, you will remain down there. I always believe in myself, I will never get carried away by whatever they say or do to me.” *

 The COVID-19 pandemic was perceived by some to be divinely decisioned analogous to perceptions about HIV in Africa. Accordingly, prayer was invoked not only as a healing remedy but to cope with the adversities of stigma and discrimination that cast a shadow on participants hope in life. Seeking divine favor through supplicatory prayers seemed logical by some participants to deal with “ignorant and evil-minded behaviors” against them. Prayer as a spiritual facet of personal coping was also manifest in how participants expressly associated their routine strength, fast recovery, and coping with labelling and stereotyping to daily devotion and renewed relationship with God (“…I keep praying to God to give me the heart to contain these comments and He does. His grace is keeping me well”). Related to prayers was seeking counselling from relatives, pastors and other people of God to gain self-control over any mishap of the COVID-19.

 Withdrawal behaviors were adopted by participants to cope with the anxiety-provoking elements of prejudices and social barriers at the workplace and the community. This took the form of being confined to home (“I have restricted myself to the house;” “it is better being indoors than being demeaned”); withdrawing from the sight of the neighborhood (“I leave for work at 4am and return late. They see you and talk about you, but if they don’t see you, can they?”);and being less responsive to calls (“I see calls and ignore them. They will claim they are checking on you, but they are not”). Other forms of coping strategies ranged from aggressive behavior, distancing to withdrawal from friends (Table 5).

**Table 5:** Coping Strategies of Adverse Stigma, Discrimination and Social Exclusion

**Adverse Experience **	**Examples of Coping Strategy**	**Coping Strategy **	**Illustrative Quote **
Stigmatization	Tenants were told to cease talking to a participant. Upon hearing this, the participant withdrew from the house cleaning roster and vowed to offend anyone who comes her way.	Distancing behavior	*“…you don’t talk to me so don’t tell me to clean. If you do then you are in for a fight.”*
	A participant responded to labelling by being aggressive to offenders including using abusive language.	Aggressive behavior	*“ Anybody who calls me names will get insults in return. I won’t take nonsense.”*
	A participant acted against stereotyping behaviors by issuing threats to offenders.	Aggressive behavior	*“I have warned people that I will not take it kindly if they call me corona.”*
Discrimination	Deliberate absenteeism was deployed by some participants to cope with workplace discrimination.	Poor commitment to work	*“There is no work for me to do so there is no need going to work all the time.”*
	Feigning sickness in order to stay off work was used to cope with undesirable behaviors at the workplace.	Poor commitment to work	*“ …if you don’t go to work, you get query. If you go to work, you are not happy. What do you do? Just pretend that you are sick and stay home.”*
	Intention to quit job in response to unfair treatment and relational barriers.	Organizational withdrawal	*“…with what is happening to me, I will leave if I get another job.”*
Social exclusion	A participant blocked contact with the close friends exhibiting socially constraining behaviors.	Social withdrawal	*“I am doing away with friends who look down on me because I tested positive.”*

## Discussion

 The first part of the findings illuminates the challenges or barriers to enforcing public health measures to curb the spread of the virus. A combination of interpersonal trust and norms embedded in social circles, workplace and home settings weakened adherence to the COVID-19 containment protocols to the extent that even if individuals visibly exhibited COVID-19 related symptoms, they were still considered as not having the virus and maintained casual contacts with others. Such practices were either the result of reckless behaviors, poor knowledge of the virus transmission process or misinformation that adherence to the protocols does not necessarily guarantee immunity from infection.^20^ This finding support studies showing that negative perceptions, social trust and poor knowledge of the COVID-19 compromised adherence to public health measures.^21,22^ Taken together, the findings highlight how control of behavioral risk factors of the COVID-19 transmission is complex because of the choices that individuals make for their own good to the detriment of the common good.^21^

 Consistent with the works of Peprah and Gyasi^9^ and Ransinget al^13^ social stigmatization in its many forms including labelling, prejudices and stereotyping demonstrated against COVID-19 survivors were built into social structures and institutions. This worked to constrain routine activities and social life of the survivors after recovery. The participants also complained about adverse behaviors that did not only undermine their status and social standing, but also discouraged personal efforts and initiative within the community structures. This is foreseen to have a long term effect of distancing survivors from community oriented programs as reported elsewhere.^23^ Generally, social stigmatization against post infected persons is typical of viral epidemics. For example, survivors of the Ebola disease in Liberia, Guinea and DR. Congo were reported to be socially devalued, received disparaging comments and subjected to social abuses.^24-26^

 Studies have highlighted the problem of secondary stigmatization against COVID-19 infected persons.^9,13^ This was also revealed in this study as participants lamented about negative attitudes and marginalization against their family members and relatives. Clearly, economic sources of livelihood among family and relatives were distorted as were needed social support systems. Secondary stigmatization was shown to be a clear manifestation of poor knowledge of the COVID-19 pathology^27^ exacerbated by the scale of infodemic across multiple channels that attracted diverse interpretations about the disease and infected people. The diffusion of COVID-19 infodemic many of which are founded on falsehood fabrications around transmission, cures and contiguity infection have generated fearful perceptions about infected populations and their close relations^28^ as mirrored in the findings. Fake information and propaganda about the COVID-19 have misled people into wrong assumptions that survivors are still contagious and therefore pose significant risk to public health and safety.^6,13^ Cultural induced stigmatization appeared in this study as reported elsewhere.^29^ This demonstrates how scientific facts of the COVID-19 are undermined by traditional leaders seeking to define and promote their own mitigation approaches against the spread of the disease.

 The participants reported experiencing discrimination ranging from the family to the community and the workplace. Of the many forms of discrimination experienced, discrimination within the family was the most disturbing given that the family has a moral obligation to provide needed social support, comfort care and interface with health professionals for better therapeutic outcomes of COVID-19 survivors.^30^ Dealing with family level discrimination is important because it promotes and perpetuates false perceptions of survivors within the neighborhood and the wider society.

 The findings pertaining to discrimination at the workplace resonate with da Silva,^31^ and builds on the literature about COVID-19 induced injustices in formal organizations. The participants reported experiencing reversed social behaviors and social injustices from coworkers and managers. Such practices constrained productivity at, and mental attachment to, work. This form of structural discrimination brings to the fore the many ways in which COVID-19 has changed social dynamics, and vertical and horizontal relations in formal organizations.^32^ The findings also points to poor institutional structures that support collegiality with, and receptivity, toward COVID-19 recovered people returning to work. Discrimination at the workplace produced counterproductive behaviors and turnover intentions as participants felt dissatisfied, demotivated and insecured at the workplace. This finding aligns with the study by Bajramiet al^33^ where COVID-19 related dynamics in organizations was reported to influence employees’ poor commitment to work and withdrawal behaviors.

 Social disconnections associated with the COVID-19 pandemic has been a challenge to building critical social capital to fight fear and misinformation. The findings demonstrate the need to be proactive in tackling this growing problem. Because, the experience of social avoidance, social neglect, and barriers to realization of social life creates a sense of despair and mental detachment from the reality of life.^5^ Nonetheless, the social barriers encountered by the participants were predicated on fear and misplaced perceptions of the COVID-19. Certain falsehood narratives about the disease across socio-ecological levels (workplace, community, and social institutions) have provoked the creation of social hierarchies apparently segregating COVID-19 infected persons from the rest of society.^23^ But it is worth noting that public health response mechanisms of the COVID-19 may have also played a role in promoting social exclusion. Whether acknowledged or overlooked, mitigation measures such as enforcement of quarantine/isolation, social distancing, mask wearing, and the prevention of large social congregations have somewhat stirred mindsets that no one is safe for casual physical contact. It is this ‘no one is safe syndrome’ that propagate othering behaviors and the feeling of discomfort being around COVID-19 infected persons.^23^

 The findings are consistent with the literature that psychosocial problems and mental instabilities are common sequela of stigmatization, discrimination and social relational barriers under the COVID-19.^5,13,34^ The experience of stigmatization was shown to be associated with reduced happiness, insomnia, anxiety disorders, fear, and low morale and self-confidence. Stigmatization on the other hand was linked to the feeling of distress, dejection, loneliness, frustration, and self-reported poor health.^9^ The perceived poor physical, emotional, and mental wellbeing of the survivors call for timely interventions to tackle the underlying risk factors. Without that, the dire consequences could be catastrophe in the short to long term.

 Participants adopted several coping strategies to overcome the dehumanizing behaviors against them. Prominent coping methods were supplicatory prayers, self-assertiveness, believing in self, withdrawal from friends and organizational withdrawal. Coping strategies in response to stigma and discrimination of infectious diseases are not new. During the Ebola outbreak in Sierra Leone, coping strategies driven by stigma and discrimination were often deployed by frontline workers and patients to manage mood, psychological problems and anxiety disorders.^24^ Similarly, HIV and mentally ill patients were shown to adopt prayers, self-assurance, and social withdrawal in managing stigma and related behaviors.^35,36^ In one breadth, coping strategies were seen as beneficial resources in alleviating the adverse experiences. On the other hand, however, the long-term effect can be detrimental than envisaged. Social withdrawal and aggressive behaviors, for example, can deprive participants of social support and capital which are needed to cope with difficulties and distress.

###  Implications of the Findings for Policy and Practice 

 In the face of the analysis, we propose interventions that can trigger behavior change and responsible behaviors toward COVID-19 survivors. Launching social campaigns to fight stigma in partnership with social influencers (revered chiefs, celebrities, athletes, health workers etc) and the media, for example, can enable society become emotionally tuned to COVID-19 survivors by embracing them within the family and community structures. Role plays code named “who are you to judge” have previously been successfully deployed to diffuse stigma and discrimination against HIV infected persons and can equally be useful in combating stereotypes and prejudices against COVID-19 survivors.

 Psychosocial support services are urgently needed to rebuild broken minds, thoughts and the disorientations experienced. Effective psychosocial support systems can bring about greater relief and activate the hopes of survivors in life. The Government’s COVID-19 response program can partner with telecom companies to develop and disseminate standardized information toolkits about the pandemic while also encouraging the general public to practice information hygiene by sanitizing, filtering and verifying information from unknown sources. Finally, privacy and confidentiality in the interface between patients and the COVID-19 management team can minimize the risk of stigma and discrimination against the former.

## Conclusion

 This study analyzed how and why COVID-19 survivors experienced and coped with stigma, discrimination, and social exclusion after recovery. The findings suggested that the sources of infection, many of which were preventable were the consequences of overlooking the protocols driven in part by the deep-rooted Ghanaian social customs of strong affinity and identity with friends and relations. We demonstrated that the disclosure of COVID-19 positive test results without accompanying psychological counseling caused significant distress and seemingly paranoid behaviors among the survivors.

 The effect of the poor disclosure process, combined with the experiences of stigma, discrimination and social avoidance negatively affected the survivors as they endured reduced mental and psychosocial well-being. Stigma, discrimination and social disapproval were shown to be driven by fear of the disease, fear of infection, panic reactions, and amplified by the diffusion of deceptive information about the COVID-19. The findings, overall, highlight that the survivors, although recovered are routinely constrained in many unfortunate ways. They appeared to be living with a “double pandemic” of having to battle for recovery from the disease in addition to fighting behavioral adversities within the societal and organizational context. The COVID-19 pandemic will eventually die out, but the sequels of stigma, discrimination and social exclusion might prevail for an unknown period and impose on survivors social and economic cost of access to medical care in future.

###  Limitations of the Study 

 There are some limitations of the study to be acknowledged. First, of the 16 Regions in the country, the study was conducted in 1 region with the highest number of cumulative and active confirmed COVID-19 cases. The transmission trajectories of the region could have partly influenced the fear and behavioral odd perceptions of COVID-19 infected persons. Further studies expanding the scope to other regions is important to enable comparison of the experience of stigma and discrimination and their sequels across regions. The data collection was limited to telephone interviews which did not allow for observation of participants in their natural environment. Future studies deploying observational data and focus group discussion where possible is recommended.

## Acknowledgements

 The authors are grateful to the COVID-19 survivors who participated in the study and provided useful information for the findings. Further appreciation goes to the case management team in the COVID-19 treatment facilities who assisted in the data collection.

## Ethical issues

 Ethical approval to conduct the study was sought from the Ghana Health Service. The study’s objective and contribution to managing the collateral effect of the COVID-19 were explained to each participant to make informed decision regarding whether to participate. Informed consent and the right of withdrawal from the interview process were also sought from each participant.

## Competing interests

 Authors declare that they have no competing interests.

## Authors’ contributions

 RAA and NMIA conceptualized the paper, collected data, and wrote the main manuscript text. AA reviewed literature, presented results and proofread the paper. All authors reviewed the final manuscript.

## References

[1] Barach P, Fisher SD, Adams MJ (2020). Disruption of healthcare: will the COVID pandemic worsen non-COVID outcomes and disease outbreaks?. Prog Pediatr Cardiol.

[2] Haider N, Osman AY, Gadzekpo A (2020). Lockdown measures in response to COVID-19 in nine sub-Saharan African countries. BMJ Glob Health.

[3] Szczesniak D, Ciulkowicz M, Maciaszek J (2020). Psychopathological responses and face mask restrictions during the COVID-19 outbreak: results from a nationwide survey. Brain Behav Immun.

[4] Wang C, Chudzicka-Czupała A, Grabowski D (2020). The association between physical and mental health and face mask use during the COVID-19 pandemic: a comparison of two countries with different views and practices. Front Psychiatry.

[5] Serafini G, Parmigiani B, Amerio A, Aguglia A, Sher L, Amore M (2020). The psychological impact of COVID-19 on the mental health in the general population. QJM.

[6] Ahinkorah BO, Ameyaw EK, Hagan JE Jr, Seidu AA, Schack T (2020). Rising above misinformation or fake news in Africa: another strategy to control COVID-19 spread. Front Commun.

[7] Fiorillo A, Gorwood P (2020). The consequences of the COVID-19 pandemic on mental health and implications for clinical practice. Eur Psychiatry.

[8] Galea S, Merchant RM, Lurie N (2020). The mental health consequences of COVID-19 and physical distancing: the need for prevention and early intervention. JAMA Intern Med.

[9] Peprah P, Gyasi RM (2021). Stigma and COVID-19 crisis: a wake-up call. Int J Health Plann Manage.

[10] Cipolletta S, Ortu MC. COVID-19: common constructions of the pandemic and their implications. J Constr Psychol. 2020:1-17. 10.1080/10720537.2020.1818653.

[11] Veldsman DP (2020). God’s spirit (of wisdom) has been sent into the world, not COVID-19: a contextual systematic-theological perspective. HTS Teologiese Studies/Theological Studies.

[12] de-Graft Aikins A (2020). ‘Colonial virus’? creative arts and public understanding of COVID-19 in Ghana. Journal of the British Academy.

[13] Ransing R, Ramalho R, de Filippis R (2020). Infectious disease outbreak related stigma and discrimination during the COVID-19 pandemic: drivers, facilitators, manifestations, and outcomes across the world. Brain Behav Immun.

[14] Mattia JG, Vandy MJ, Chang JC (2016). Early clinical sequelae of Ebola virus disease in Sierra Leone: a cross-sectional study. Lancet Infect Dis.

[15] Statista. Coronavirus cases in Africa as of April 22, 2021, by country. Hamburg, Germany: Statista GmbH; 2021.

[16] Govender K, Cowden RG, Nyamaruze P, Armstrong RM, Hatane L (2020). Beyond the disease: contextualized implications of the COVID-19 pandemic for children and young people living in Eastern and Southern Africa. Front Public Health.

[17] Semo BW, Frissa SM (2020). The mental health impact of the COVID-19 pandemic: implications for sub-Saharan Africa. Psychol Res Behav Manag.

[18] Ghana Health Service. COVID-19: Ghana outbreak response management updates. 2021. https://www.ghanahealthservice.org/covid19/archive.php. Accessed February 6, 2020.

[19] Guest G, Bunce A, Johnson L (2006). How many interviews are enough? an experiment with data saturation and variability. Field Methods.

[20] Coetzee BJ, Kagee A (2020). Structural barriers to adhering to health behaviours in the context of the COVID-19 crisis: considerations for low- and middle-income countries. Glob Public Health.

[21] Widayati A. Knowledge, perceptions, and awareness related to COVID-19 among the Indonesian adults during the outbreak’s escalation period: a cross-sectional online survey in Yogyakarta province, Indonesia. Asia Pac J Public Health. 2021:10105395211001655. 10.1177/10105395211001655. 33729018

[22] Nivette A, Ribeaud D, Murray A (2021). Non-compliance with COVID-19-related public health measures among young adults in Switzerland: insights from a longitudinal cohort study. Soc Sci Med.

[23] Bhattacharya P, Banerjee D, Rao TS (2020). The “untold” side of COVID-19: social stigma and its consequences in India. Indian J Psychol Med.

[24] McMahon SA, Ho LS, Brown H, Miller L, Ansumana R, Kennedy CE (2016). Healthcare providers on the frontlines: a qualitative investigation of the social and emotional impact of delivering health services during Sierra Leone’s Ebola epidemic. Health Policy Plan.

[25] O’Leary A, Jalloh MF, Neria Y (2018). Fear and culture: contextualising mental health impact of the 2014-2016 Ebola epidemic in West Africa. BMJ Glob Health.

[26] Keita MM, Taverne B, Sy Savané S (2017). Depressive symptoms among survivors of Ebola virus disease in Conakry (Guinea): preliminary results of the PostEboGui cohort. BMC Psychiatry.

[27] Parasher A (2021). COVID-19: current understanding of its pathophysiology, clinical presentation and treatment. Postgrad Med J.

[28] Dash S, Parray AA, De Freitas L (2021). Combating the COVID-19 infodemic: a three-level approach for low and middle-income countries. BMJ Glob Health.

[29] Okereke M, Ukor NA, Ngaruiya LM (2020). COVID-19 misinformation and infodemic in rural Africa. Am J Trop Med Hyg.

[30] Hart JL, Turnbull AE, Oppenheim IM, Courtright KR (2020). Family-centered care during the COVID-19 era. J Pain Symptom Manage.

[31] Teixeira da Silva JA (2020). Stigmatization, discrimination, racism, injustice, and inequalities in the COVID-19 era. Int J Health Policy Manag.

[32] Kniffin KM, Narayanan J, Anseel F (2021). COVID-19 and the workplace: implications, issues, and insights for future research and action. Am Psychol.

[33] Demirović Bajrami D, Terzić A, Petrović MD, Radovanović M, Tretiakova TN, Hadoud A (2021). Will we have the same employees in hospitality after all? the impact of COVID-19 on employees’ work attitudes and turnover intentions. Int J Hosp Manag.

[34] Singh R, Subedi M (2020). COVID-19 and stigma: social discrimination towards frontline healthcare providers and COVID-19 recovered patients in Nepal. Asian J Psychiatr.

[35] Ironson G, Kremer H, Lucette A (2016). Relationship between spiritual coping and survival in patients with HIV. J Gen Intern Med.

[36] Rzeszutek M (2018). Health-related quality of life and coping strategies among people living with HIV: the moderating role of gender. Arch Womens Ment Health.

